# New Developments and Applications of the MP2RAGE Sequence - Focusing the Contrast and High Spatial Resolution R_1_ Mapping

**DOI:** 10.1371/journal.pone.0069294

**Published:** 2013-07-16

**Authors:** José P. Marques, Rolf Gruetter

**Affiliations:** 1 Department of Radiology, University of Lausanne, Lausanne, Switzerland; 2 Laboratory for Functional and Metabolic Imaging, Ecole Polytechnique Fédérale de Lausanne, Lausanne, Switzerland; 3 Department of Radiology, University of Geneva, Geneva, Switzerland; University of Minnesota, United States of America

## Abstract

MR structural T1-weighted imaging using high field systems (>3T) is severely hampered by the existing large transmit field inhomogeneities. New sequences have been developed to better cope with such nuisances. In this work we show the potential of a recently proposed sequence, the MP2RAGE, to obtain improved grey white matter contrast with respect to conventional T1-w protocols, allowing for a better visualization of thalamic nuclei and different white matter bundles in the brain stem. Furthermore, the possibility to obtain high spatial resolution (0.65 mm isotropic) R_1_ maps fully independent of the transmit field inhomogeneities in clinical acceptable time is demonstrated. In this high resolution R_1_ maps it was possible to clearly observe varying properties of cortical grey matter throughout the cortex and observe different hippocampus fields with variations of intensity that correlate with known myelin concentration variations.

## Introduction

The promise of ultra high fields systems to provide higher spatial resolution structural images due to their higher signal to noise ratio (SNR) has been hampered by the increase in transmit magnetic field inhomogeneities and the high specific absorption rate (SAR) that come associated with the decrease of the wavelength and increased frequency of the RF pulses that are used to excite the proton spins [1]. SAR limitations have made gradient echo based sequences to be the main workhorse of structural imaging at high fields, delivering eitherT_2_^*^-weighted imaging [2], phase imaging [3] and T_1_-weighted imaging. The MP2RAGE sequence has been recently introduced as a means to obtain bias field free T_1_-weighted images and jointly estimating T_1_ maps at ultrahigh field [4], [5]. In the original work, the sequence parameter optimization was developed to obtain the conventional range of contrast used in T_1_-weighted imaging (covering the T_1_ range from white matter to cerebro spinal fluid) with the protocols being defined in order to achieve reliable T_1_ maps when the typical clinical whole brain isotropic resolution of approximately 1 mm was desired. Although such a large range of T_1_ range and conservative resolution is desirable for normal brain imaging and segmentation applications, it is not ideal when looking at detailed visualization of deep gray matter structures [6] or fine variations in cortical relaxation properties throughout the brain [7].

Visualization of deep gray matter structures such as the different thalamus nuclei, striatum, external and internal globus pallidus (GPe/GPi), red nucleus (RN) and substantia nigra (SN) can be of great importance in applications such as deep brain stimulation (DBS) used in the treatment of involuntary movement disorders, in Parkinson’s disease or dystonia [8], [9]. Attempts to automatically segment the thalamus nuclei using structural imaging have to date had some success [10], [11], but only limited correlation was found with thalamic segmentation using either diffusion weighted imaging [12] or histology. Due to the low levels of contrast between the basal ganglia and surrounding structures in T_1_-w images, a number of approaches using other contrast have been explored [10], [13]–[15]. Among these, a recent studies have proposed a modification of the standard MPRAGE sequence parameters to yield a better visualisation of basal ganglia structures [16] and subthalamic nuclei [17] by using a shorter inversion time than the usually used at the respective field strengths (in which the CSF signal is nulled). Recently in a 3T study, the MP2RAGE was modified [18] in order to provide two T_1_-weighted images, one with the conventional MPRAGE contrast (with CSF nulling) and one with a short TI where the WM signal is suppressed.

Despite the optimization of the MP2RAGE sequence parameters [5] performed in order to reduce B_1_^+^ dependence of those images (at a cost of a reduction of the contrast obtainable), the resulting T_1_-maps still suffer from some residual transmit field bias. Furthermore, the temptation to increase the resolution (by increasing the number of low flip angle excitations per TR) and the need to keep the total acquisition time low (by reducing the TR of the MP2RAGE), increase the sensitivity of the MP2RAGE T_1_ estimation to B_1_^+^ inhomogeneities. Recently, some attention has been drawn to the correlation between the observed cortical T_1_ values and known distributions of myelination [7], [19], [20]. For these correlations to be further evaluated at such high resolution and through such a large brain extent, it is imperative to obtain high resolution, robust and fully bias free T_1_ values. Often, the effect of T_1_ relaxation and B_1_^+^ inhomogeneity on signal are intertwined and most techniques proposed to measure T_1_ effectively measure these T_1_ and B_1_^+^ simultaneously [21]–[26]. This can be done either within one single acquisition, which impacts on the SNR efficiency of the method, or using separate acquisitions.

In this work, we explore different potential high field applications of the MP2RAGE sequence: (i) improved contrast between white matter and grey matter tissues allowing clear visualization of sub-thalamic nuclei and other deep grey matter structures; (ii) high resolution T_1_ maps with optimum contrast to noise ratio in which the effects of significant B_1_ field inhomogeneities are taken into account by combining the MP2RAGE sequence with the Sa2RAGE sequence [27] and study R_1_ variations throughout the cortex and in different white matter bundles.

## Theory and Methods

The MP2RAGE sequence can be described as an inversion recovery sequence in which two gradient echo images are acquired during the recovery period with different inversion times (GRE_TI1_ and GRE_TI2_). The MP2RAGE image is a synthetic image obtained from a combination of the two acquisitions:
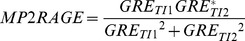
(1)both images are taken as complex images and the asterisk stands for the complex conjugate.

**Figure 1:**
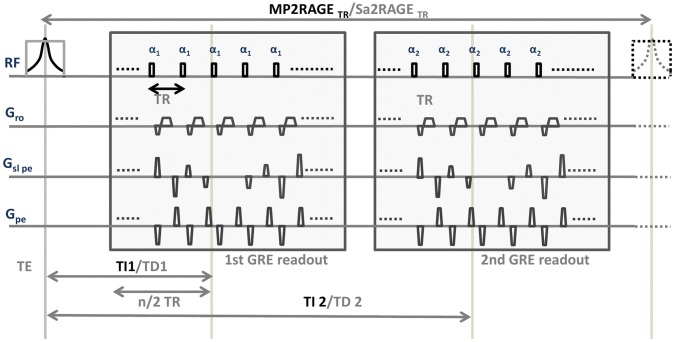
Diagram of the MP2RAGE and Sa2RAGE sequences. Inversion (Delay) times TI1 and TI2 (TD1 and TD2) are defined as the time from the middle of the inversion (saturation) pulse to the excitation corresponding to the center k-space line in the phase encoding in the slab selection direction. MP2RAGETR is the time between two successive inversion pulses and TR is the time between successive excitation pulses in the GRE kernel which is composed of n excitations. Features specifically associated with the MP2RAGE and Sa2RAGE are shown in black and light grey respectively (the adiabatic inversion and the saturation pulse). The gradient echo excitation pulses used for both the SA2RAGE and MP2RAGE were non-frequency selective.

The Sa2RAGE sequence is a saturation recovery sequence in which two images are acquired before (GRE_TD2_) and after (GRE_TD1_) a saturation pulse (see Figure 1 for a more complete description). A full Bloch equation simulation of the sequence permits the mapping of the transmit field B_1_. It was shown [27] that this technique could provide B_1_ maps at 7T with small errors even when assuming a common T_1_ value for the whole brain.

### Simulations

The optimum sequence parameters for the different applications were studied via simulations. The predicted MP2RAGE signal amplitudes for several tissues were numerically calculated after solving the Bloch Equations as in reference [5].

When studying the (i) optimum contrast between white matter WM, thalamus and Gray Matter the following parameters were considered fixed: number of excitations per GRE module was set to 160 (full k-space coverage) or 120 (partial fourier k-space coverage); The contrast at 7 Tesla was evaluated for 5 different T_1_ values, ranging from 1.1 (∼T_1WM_) to 1.9s(∼T_1GM_) [5]. The range of T_1_ values chosen should cover the different T_1_ values in the thalamus and the choice of the number of excitations was done to allow a 1mm isotropic resolution with full brain coverage in sagittal orientation.

When studying the (ii) optimum contrast achievable at high resolution (0.65 mm isotropic): Number of excitations per GRE module was set to 192 (partial fourier k-space coverage 6/8–256 partitions); 5 T_1_ values equally spaced ranging from 1.1 (∼T_1WM_) to 4 s (∼T_1CSF_). The range of T_1_ values was chosen in order to include all brain tissues and CSF, while the choice of the number of excitations was done to achieve a 0.65 mm isotropic resolution with full brain coverage in sagittal orientation.

In both cases, the following parameters were varied in order to compute the optimum protocols: MP2RAGE_TR_, TI_1_ and TI_2_, α_1_ and α_2_ (see Figure 1 for a complete overview of the sequence and the meaning of the different parameters).

Contrast to noise by unit of time between two tissues (i and j) with successive values of T_1_ was defined as:
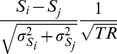
(2)


Where S_i_ is the calculated MP2RAGE signal [5] of tissue i, and its noise, σ_Si_, was estimated by error propagation of the MP2RAGE signal equation. The contrast to noise ratio between the successive T_1_ values was integrated in order to obtain the sequence CNR for the desired application.

Sequence parameters were chosen from the simulations in order to optimize the CNR for the desired T_1_ range and for the desired resolution. A ground truth T_1_ mapping protocol was designed to evaluate the accuracy of the T_1_ correction methodology (experiment ii). The ground truth protocol consisted of an MP2RAGE acquisition with a reduced number of rf pulses during the recovery process (hence, to keep the FOV the same, a lower resolution was used) and reduced flip angle amplitude for both echo trains (ensuring the low B_1_^+^ inhomogeneity sensitivity). In this protocol, the maximum achievable CNR was penalized in order to have a bias in the T_1_ mapping under 5% for all tissues in the brain when in the presence of B_1_ field deviations as large as ±40% of the nominal value.

### Experimental Protocol

Data were collected at a short-bore 7T MR system (Siemens, Germany) equipped with a head-gradient insert and a 32-channel head coil (Nova Medical Inc) for reception. All subjects provided written informed consent and the study was approved by the local ethics committee (Commission cantonale VD d’éthique de la recherche sur l’être humain).

(i) MP2RAGE data from 3 subjects (age = 24±4 years) were acquired using the following sequence parameters:

- MP2RAGE_TR_/TI_1_/TI_2_ = 6/0.8/2.5 s and α1/α2 = 4/5 degrees (Protocol A);
- optimized for the white to grey matter T_1_ range MP2RAGE_TR_/TI_1_/TI_2_ = 6/0.7/1.6 s and α1/α2 = 7/7 degrees (Protocol B).

Both acquisitions were performed using iPAT_PE_ = 2 and 6/8 k-space coverage on the slice encoding direction, acquisition time of 10 mins. The matrix size and resolution were of 256×200×176 and 0.85 mm isotropic respectively, while the BW was of 240 Hz per pixel.

(ii) Data from 4 subjects (age = 28±4 years) were acquired using the following sequences:

- MP2RAGE sequence: MP2RAGE_TR_/TI_1_/TI_2_ = 6/0.8/2.7 s, α_1_/α_2_ = 7/5 degrees (protocol A - high CNR), matrix size and resolution were of 320×320×256 and 0.65 mm isotropic, T_acq_ = 10min, iPAT_PE_ = 3 and 6/8 k-space;
- MP2RAGE sequence: MP2RAGE_TR_/TI_1_/TI_2_ = 6/0.8/2.7 s, either α_1_/α_2_ = 3/4 (protocol B - reduced B_1_^+^sensitivity), BW = 300 Hz per pixel, matrix size and resolution were of 192×192×160 and 1 mm isotropic, T_acq_ = 10 min.26 secs;
- Sa2RAGE sequence: Sa2RAGE_TR_/TD_1_/TD_2_ = 2.4/0.058/1.8 s, α_1_/α_2_ = 4/11 degrees, TR_GRE_ = 3 ms, BW = 1950 Hz per pixel, matrix size and resolution were of 128×120×64 and 2×2×2.5 mm^3^ resolution, iPAT_PE1_ = 2 and 6/8 partial Fourier sampling were used in the phase encoding direction and slice encoding direction respectively. T_acq_ = 2.30 min (see Fig. 1 for more details regarding the sequence);

The whole scan was repeated for one of the subjects with dielectric pads [28], [29] positioned on one side of the head to guarantee that a different B_1_^+^ pattern would be present.

### Processing Protocol (i)

**Figure 2:**
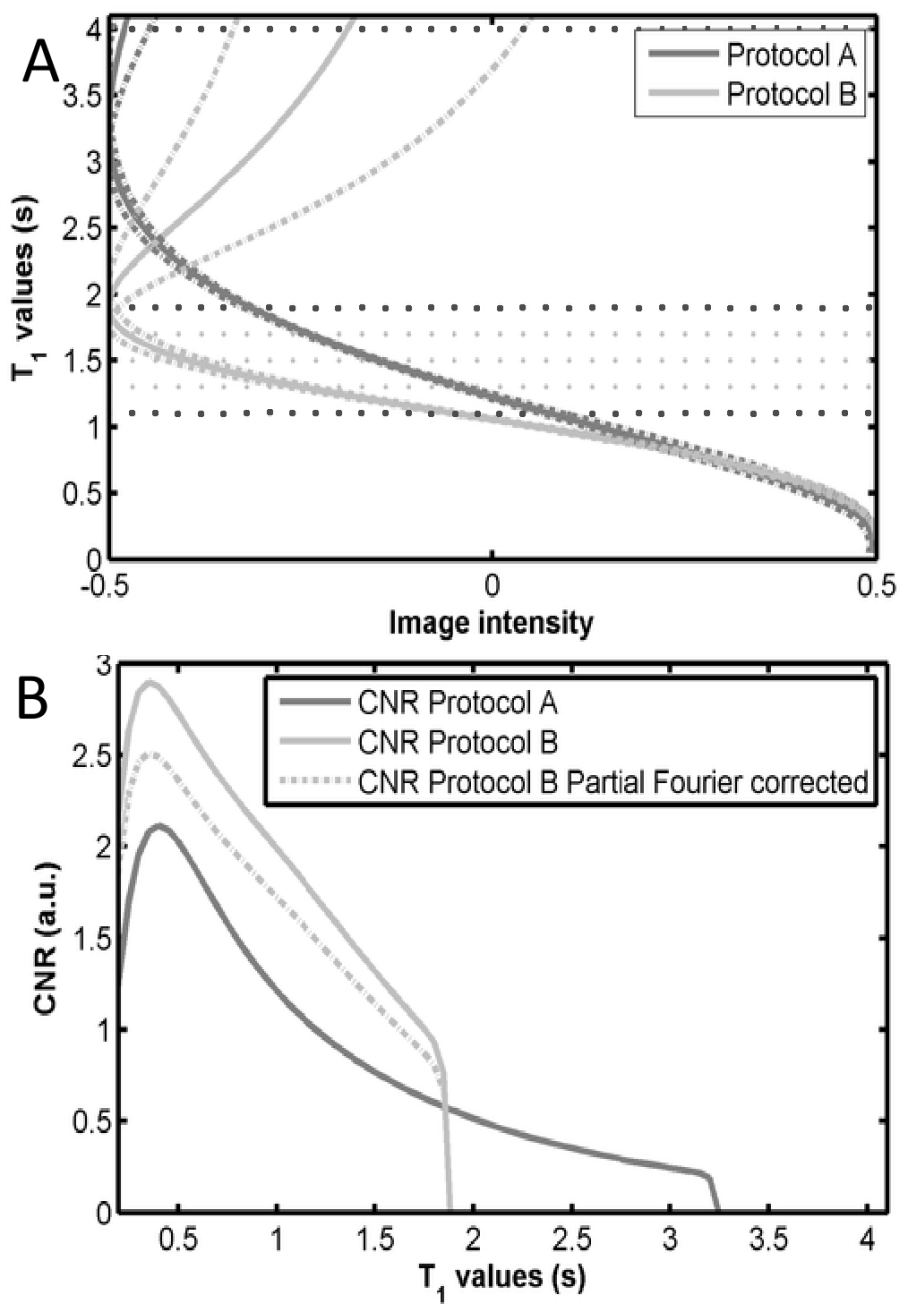
Plots of the theoretical relationship between T_1_ values and MP2RAGE intensity. (a) MP2RAGE as a function of T_1_ for the parameters that optimize contrast over all values of T_1_ present in the brain (dark grey line) and the parameters that optimize contrast for T_1_ values between those of WM and GM (light grey). The dashed lines represent the variation in intensities when the effective B_1_^+^ is ±40% of the nominal value while dark dotted lines show the T_1_ values for which Protocol A was optimised and light dotted lines show the T_1_ values for which Protocol B was optimised. (b) Plot of the contrast to noise ratio between successive T_1_ values (δT_1_ = 0.05 s) for the different protocols as a function of T_1_. The dashed line shows the corrected CNR when the effect of the Partial Fourier in protocol B is taken into account.

When performing the optimization of the MP2RAGE sequence parameters to enhance the contrast for a limited range of T_1_ values (from 1.1 to 1.9 s), it is possible to observe (see Fig. 2a), that the relationship between T_1_ and MP2RAGE signal intensity is no longer monotonous. The CSF intensity appears aliased to intensity values close to that of WM. Using the Bloch simulations it is possible to recover the full range by using information from a complex ratio between the second and first inversion time images [4], which is monotonic as a function of T_1_. Although this new image, MP2RAGE_WMGMFullRange_, has exactly the same contrast properties as the MP2RAGE_WMGM_, the fact that CSF appears dark makes the interpretation of the images easier.

Using the sequence parameters suggested to enhance sub-thalamic contrast, the intensity of WM and of CSF are close to zero in the first and second inversion contrasts respectively (note that in Fig. 2a both have an MP2RAGE intensity of ∼0). This is a similar scenario to that observed in the FLAWS sequence, where this feature was used to create Double Inversion Recovery like images [18]. Because of the specificities of the receive and transmit field inhomogeneities observed at 7T, the MP2RAGE MIP (minimum intensity projection) images were computed as:
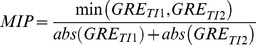
(3)


To obtain a quantitative evaluation of the contrast obtained using the different sequence parameters, regions of interest were defined on the images of protocol A (MP2RAGE) and protocol B (MP2RAGE_WMGM_) using MRIcro [30] and their intersection was used to evaluate the mean and standard deviation of the signal. The contrast to noise ratio between different structures was quantified as in Eq. 2.

### Processing Protocol (ii)

The Sa2RAGE image (ii-c) and the low resolution MP2RAGE image (ii-b) were co-registered to the high resolution MP2RAGE image using FLIRT (www.fmrib.ox.ac.uk/fsl). The co-registeration was performed using the images with higher signal intensity and lower contrast (2^nd^ contrast from the Sa2RAGE and MP2RAGE sequences) and the spatial transforms were subsequently applied to the synthetic images. Re-sampling was performed using a sinc interpolation.

**Figure 3:**
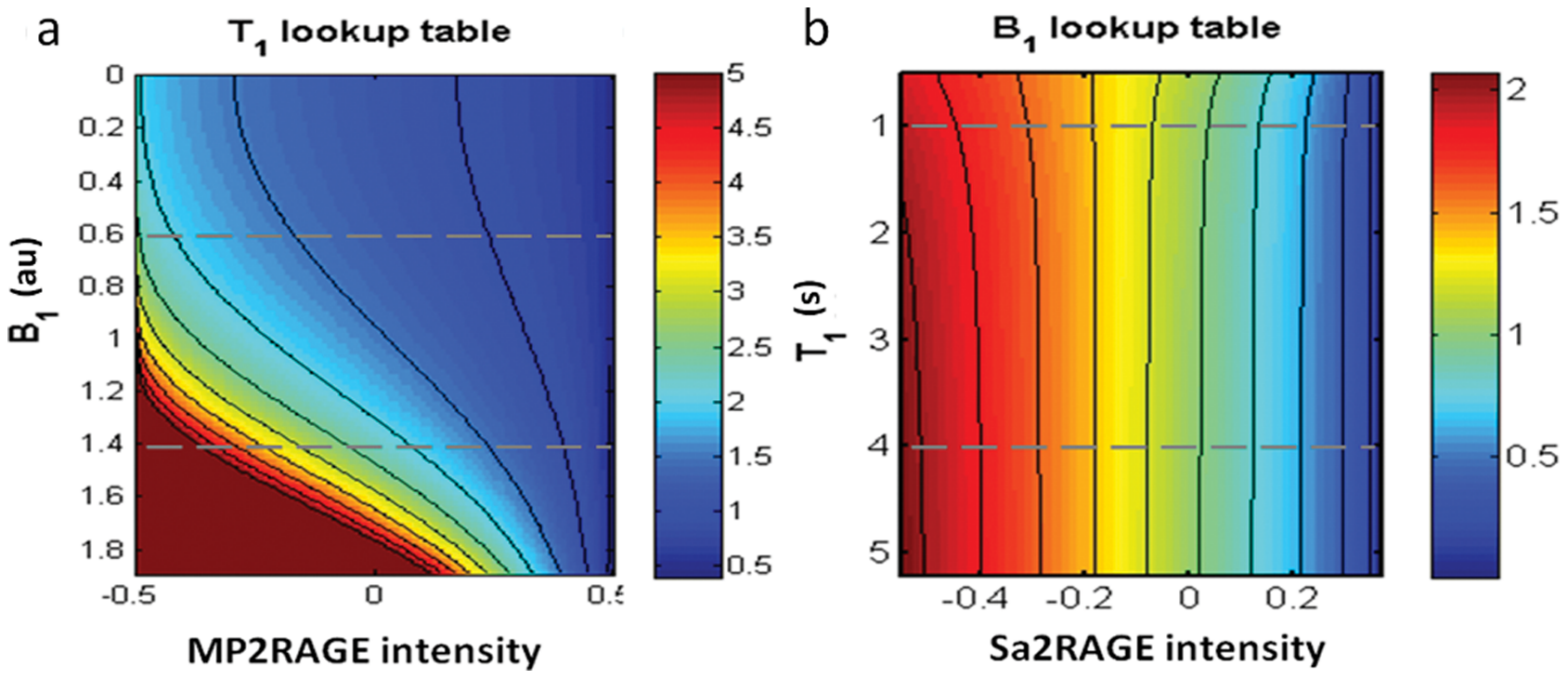
Lookup tables used to compute: (a) the R_1_ (1/T_1_) maps for the MP2RAGE sequence with MP2RAGE_TR_/TI_1_/TI_2_ = 6/0.8/2.7 s, α_1_/α_2_ = 7/5 (protocol ii-A), (b) the B_1_ maps for the Sa2RAGE sequence with Sa2RAGE_TR_/TD_1_/TD_2_ = 2.4/0.058/1.8 s, α_1_/α_2_ = 4/11. Grey dashed lines define the typical range of B_1_ and T_1_ observed in the human brain at 7T (±40% of the nominal B_1_ value).

In the original MP2RAGE T_1_ map calculation [5], look up tables were used to, assuming the transmit field was equal throughout the whole image, relate the MP2RAGE intensity to a T_1_ value. A similar process was used to calculate B_1_^+^ maps using the Sa2RAGE sequence [27], where an average T_1_ value was assumed to be valid throughout the brain and was demonstrated to have a reduced impact in terms of the robustness of the calculated B_1_^+^ maps. In this work, 2D lookup tables containing the T_1_ values associated to certain MP2RAGE signal and B_1_^+^ value (see Fig. 3a for the MP2RAGE protocol A) and the B_1_ values associated to certain Sa2RAGE signal and T_1_ value (see Fig. 3b referring to the Sa2RAGE protocol) were computed. A two dimensional interpolation was iteratively performed for each pixel using the two lookup tables. Given the higher independence of B_1_^+^ estimation on the T_1_ values (see Fig. 3b), the B_1_^+^ was first calculated for each pixel assuming constant T_1_ throughout the brain (1.5 s). These B_1_^+^ values were then used to estimate the T_1_ values via a 2D interpolation of the MP2RAGE lookup table (Fig. 3a). The process was repeated using the newly updated T_1_ estimates for each voxel. At the third iteration, the variations in both B_1_^+^ and T_1_ were found to be under 10^−3^.

To evaluate the effectiveness of the B_1_ correction, the corrected R_1_ maps (1/T_1_) acquired with the high-resolution (and high CNR) protocol (ii-A) were compared with the low resolution (and low B1-sensitivity) protocol (ii-B) in terms of general similarity between the distribution of R_1_ values throughout white and grey matter.

The quality of the anatomical information present in high resolution R_1_ maps was evaluated by observing the contrast and anatomical detail observed in structures such as the hippocampus. The individual R_1_ maps were processed with freesurfer (http://surfer.nmr.mgh.harvard.edu/) to create cortical surface models [31]. Five equally spaced surfaces were generated in between the white matter and pial surfaces in order to study cyto-architectonic cortical variations in the mid layer throughout the brain. A 2 mm smoothing along the surface was applied and the R_1_ surface maps were then plotted using MATLAB (The MathWorks Inc.).

## Results

Simulations showed that the parameters that optimise T_1_ contrast between WM and GM are obtained by reducing the spacing between the two different inversion times (see experimental Protocol i-b). Figure 2a shows the lookup tables of the MP2RAGE signal intensity as a function of the T_1_ values for the protocols with TR = 6 secs optimized for full T_1_ range contrast or WM-GM contrast (see Fig. 2a). Using partial k-space sampling in the slice encoding direction it was possible to reduce the number of excitations per GRE block and the sensitivity of the resulting image to transmit B_1_ field inhomogeneities (note that in Protocol A α1/α2 = 4/5 while Protocol B α1/α2 = 7/7) (see dashed lines in Fig. 2a). Figure 2b shows the computed CNR between two tissues of successive T_1_ values (spacing of 0.05 secs), as a function of T_1_ for the different protocols. The CNR of the new sequence parameters for the range of T_1_ values for which the optimization was performed (corresponds to the integral of the curves in Fig. 2b from 1.1 to 1.9 s) increased by 51%, which when taking into account the reduced number of excitations due to partial Fourier (PF) sampling 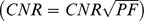 is of ∼33% (see Fig. 2b).

**Figure 4:**
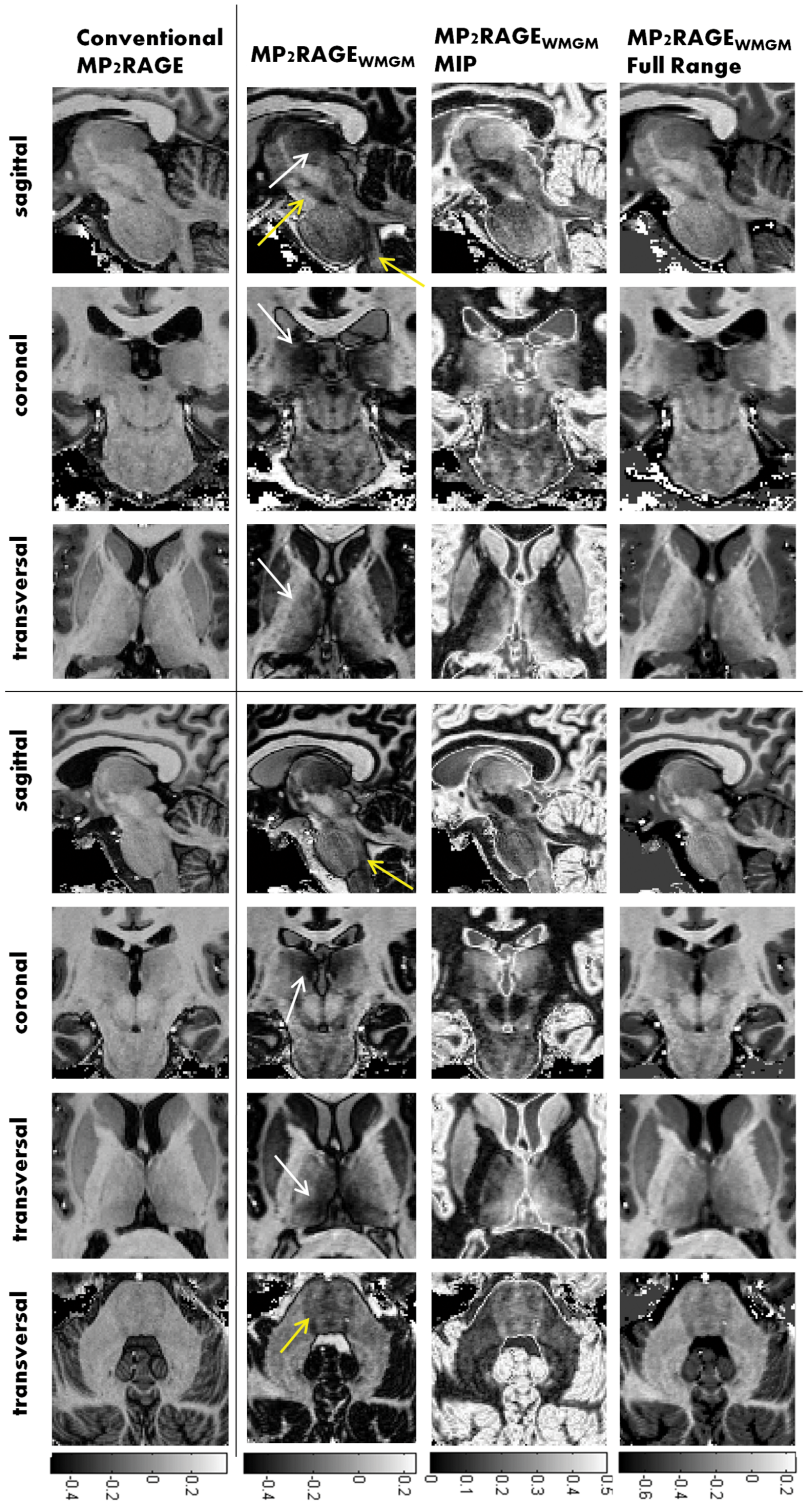
Panels with representative data from the two different subjects (Subject 1 - top three rows, Subject 2 -bottom 4 rows). The different columns show:(1^st^ column) conventional MP2RAGE; (2^nd^ column) MP2RAGE optimized for WM GM contrast;(3^rd^ column) MP2RAGE MIP images and (4th column) full range MP2RAGE_WMGM_images. Yellow arrows point regions where the increased WM T1 sensitivity of the new protocol allows the visualization of brain stem and pons sub-structures, while white arrows show the delineation the medio-dorsal, ventral lateral and pulvinar nuclei of the thalamus.

Figure 4 shows midbrain MP2RAGE and MP2RAGE_WMGM_ images. It is possible to see an increased delineation of the thalamus and its medio dorsal, ventral lateral and pulvinar nuclei (white arrows) as well as increase contrast within the brain stem (yellow arrows). It should be noted that the reduced intensity (darker MP2RAGE intensity) observed in cortical grey matter and deep brain structures does not reflect a reduction in SNR in those regions but simply the fact that the two images (GRE_TI1_ and GRE_TI2_) have similar amplitudes but opposite phases (see Fig. 2a). Table 1 shows contrast to noise values (calculated as in Eq. 2) between different regions of interest: Medial Thalamus vs outer thalamus; outer thalamus vs WM; Caudate vs the head of the Putamen; Brain Stem vs cerebellum white matter; on average the increase of the contrast to noise ratio increased by 34%, in good agreement with what was expected from simulations. Naturally, the MIP and Full Range image have contrast to noise properties that, from a quantitative perspective, are equivalent to those of MP2RAGE_WMGM_, (see Table 1). The main differences are the easier interpretability of the full range image (4^th^ column of Fig. 4) due to its conventional appearance (dark CSF and bright WM), while the MIP image (3^rd^ column of Fig. 4), having a similar appearance to Double Inversion recovery images naturally allow a tighter image display dynamic range.

**Table 1:** Table showing the mean contrast to noise ratio between different brain regions with T_1_ values in between that of cerebral white matter and cortical grey matter.

Contrast to Noise Ratio	MP2RAGE	MP2RAGE_WMGM_	MIP	MP2RAGE_FULLRANGE_
Medial Thalamus (1.4±0.1 cm^3^) vs Outerthalamus (3.2±1.5 cm^3^)	2.5±0.2	3.0±0.5	2.8±0.4	3.0±0.5
Outer Thalamus (3.2±1.5 cm^3^) vs White Matter(163±73 cm^3^)	1.9±0.5	2.5±0.7	2.6±0.7	2.5±0.7
Caudate (2.1±0.6 cm^3^) vs Putamen(1.7±0.1 cm^3^)	0.5±0.2	0.7±0.4	0.7±0.4	0.7±0.4
Brain Stem (3.3±0.7 cm^3^) vs Cerebellum whitematter (3.6±1.5 cm^3^)	1.5±0.4	2.4±0.2	2.3±0.2	2.4±0.2

The contrast to noise ratio was evaluated on the same regions of interest for the 2 different sequences (and 4 different contrasts). The mean volume of each region of interest is shown in the left column in parenthesis in cm^3^.

**Figure 5:**
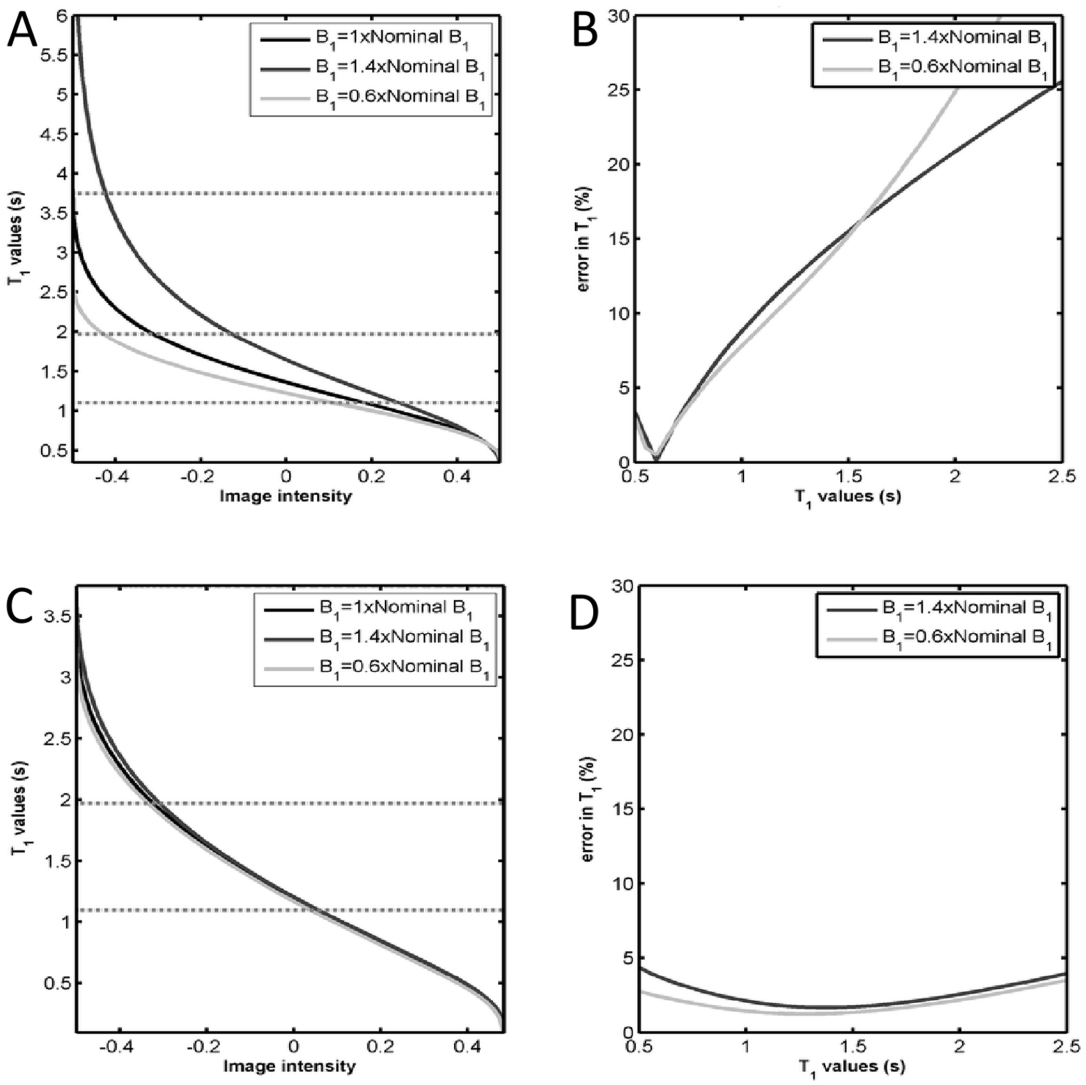
Plots of the look up tables (a,c) of the MP2RAGE intensity as a function of the T1 values and the associated error (b,d) on the T_1_ calculation as a function of T_1_ when in the presence of B_1_ values that are ±40% different from the nominal value (light and dark gray respectively). Protocols A and B are shown in the top (a,b) and bottom panel (c,d) respectively.

Figure 5 shows lookup tables associated with different protocols (ii.a and ii.b, see Methods section for more details) for 3 different relative B_1_^+^ intensities (0.6, 1 and 1.4 times the nominal B_1_). The increased B_1_^+^ sensitivity of the protocol in Fig. 5a (ii.a) is related to the increase of the number of rf pulses used per GRE block and of their amplitude (one direct and one indirect consequence of increasing the spatial resolution of the T_1_ maps and wanting to achieve optimum contrast). The protocol shown as insensitive to B_1_^+^ has errors on the T_1_ estimation that are always lower than 2.5% of the nominal value (for the range of T_1_ values expected to be found in the brain at 7T) even in the presence of ±40% deviations from the nominal B_1_^+^(see Fig. 5d). Such low sensitivity to the B_1_^+^ inhomogenity (before any B_1_^+^ correction) makes it a valuable protocol to validate the results achieved with the B_1_^+^ correction procedure.

**Figure 6:**
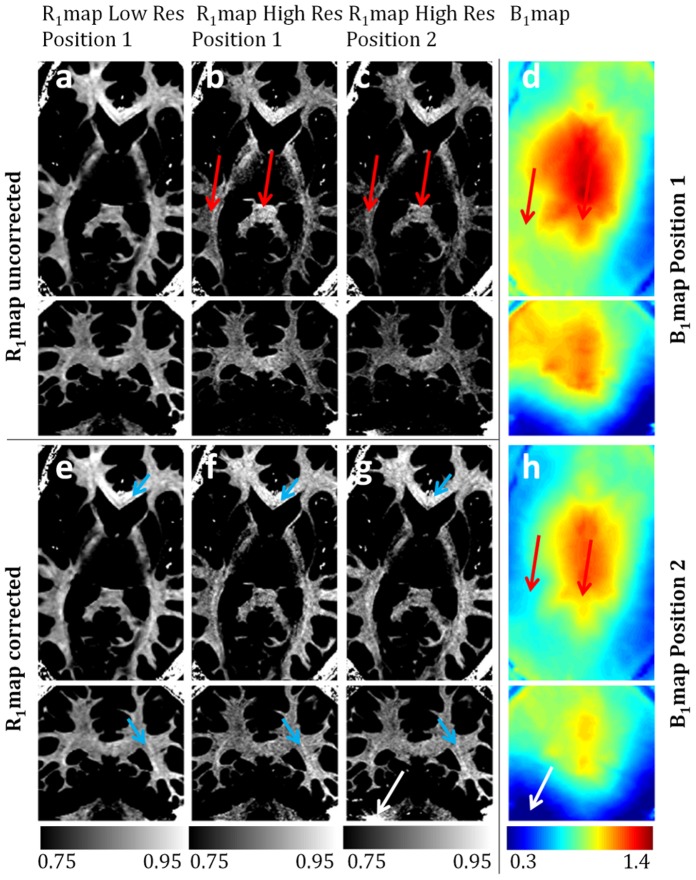
Transverse and coronal slices of: (a) Uncorrected R_1_ maps using protocol ii-B; (b,c) Uncorrected R_1_ maps using using protocol ii-A with two different head and dielectric pad positions; (e) Corrected R_1_ maps using protocol ii-B; (f,g) Corrected R_1_ maps using using protocol ii-A with two different head and dielectric pad positions; (e,h) B_1_^+^ maps corresponding to the two different head and dielectric pad positions; R_1_ maps are shown in a short range from 0.75 to 0.95 s^−1^ to emphasize the sensitivity of the R_1_ maps to the B_1_ in-homogeneity, and the success of its correction. Arrows point out regions of increased difference between in the B_1_^+^ maps that have clear implications on the uncorrected high resolution R_1_ maps. Blue arrows point out white matter fibber bundles with increased R_1_ values with respect to the remaining white matter. Red arrows point out regions of significant R_1_ differences from the ground truth prior to the B_1_^+^ correction, which are successfully corrected. The white arrow points out a region of very low B_1_^+^ field where the adiabatic condition was not reached and hence the R_1_ values are largely overestimated and not possible to correct with the proposed methodology.

Figure 6 shows different slices of R_1_ maps of one subject (whose range 0.75–0.95 s^−1^ was adapted to enhance the variations observed within white matter) obtained with two different imaging protocols and at two different head and dielectric pads [28], [29] positions (in order to create different B_1_ interference patterns). The B_1_^+^ maps, spatially co-registered to the R1 maps, are also shown (Fig. 6d,h) in a short range of relative B_1_ values (0.3–1.4). It is possible to see that the major changes between the two B_1_ maps are located in the parietal and cerebellum regions. Red arrows show regions where the spatial inhomogeneities observed in the uncorrected R_1_ maps (Fig. 6b,c) were spatially correlated with low B_1_ regions (in the parietal area) or high relative B_1_ (increased contrast observed in the splenium corpus callosum). After correction, the high resolution R_1_ maps (Fig. 6f,g) have increased similarity to the low resolution R_1_ maps (Fig. 6a,e), with a more homogeneous and symmetric distribution of R_1_ values throughout the brain. Regions where the B_1_ was too low to achieve the adiabatic condition in the bottom of the cerebellum (white arrow) cannot be corrected with the proposed methodology. Note that the low resolution R_1_ maps (Protocol ii-B) remain mostly unchanged before (Fig. 6a) and after correction (Fig. 6e), supporting its insensitivity to B_1_ inhomogeneities. Highly myelinated white matter fibber bundles such the optic radiation and the Genu corpus callosum are enhanced with respect to the remaining white matter (blue arrows) even after correction.

**Figure 7:**
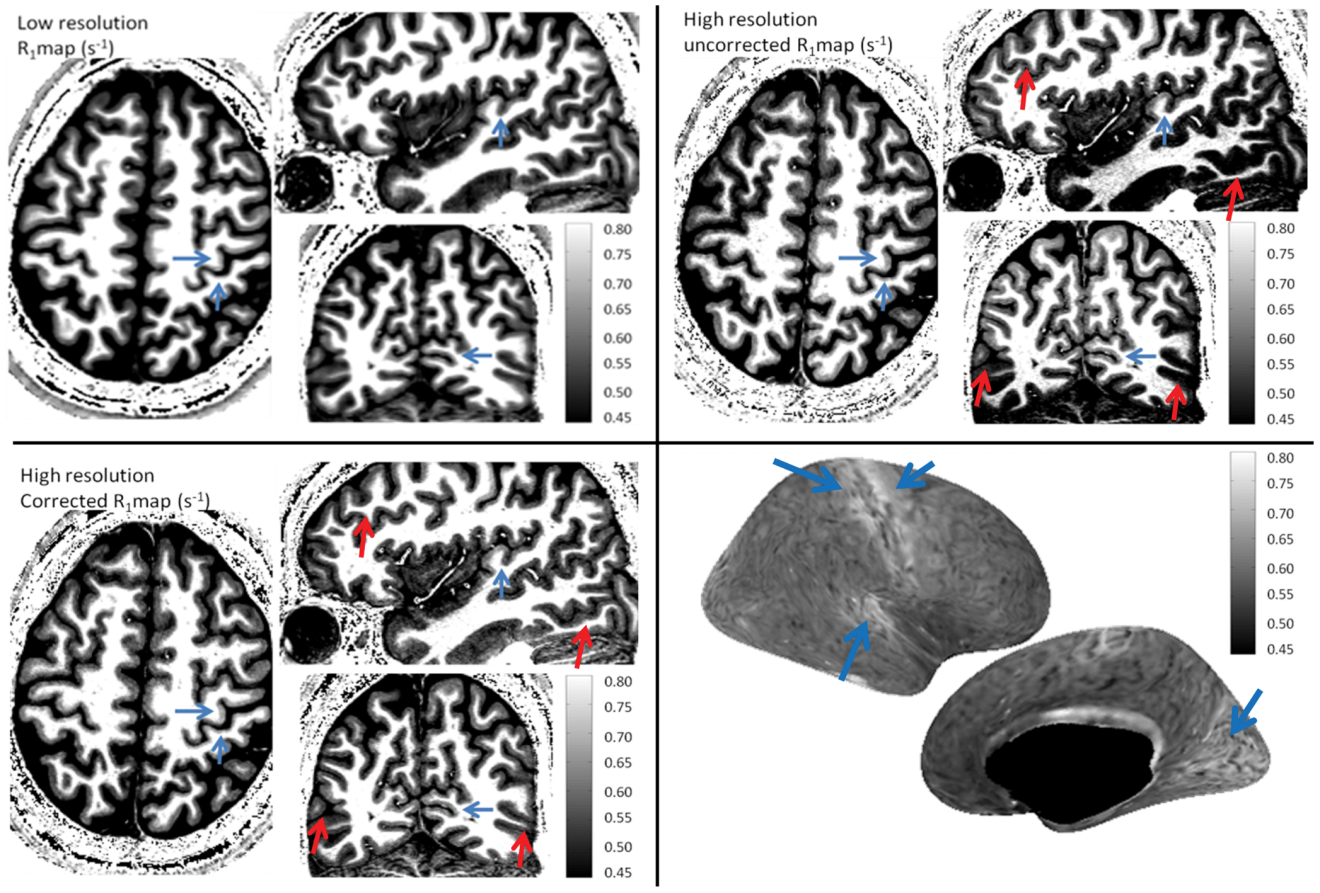
Transverse, sagittal and coronal slices of: (a) a corrected R_1_ map using protocol B and (b,c) uncorrected and corrected R_1_ maps calculated using protocol A. The R_1_ maps are shown in a short range from 0.45 to 0.80 s^−1^ in order to emphasize the sensitivity of the cortical R_1_ maps to the B_1_ in-homogeneity. Panel (d) shows the reconstructed corrected R_1_ surface across the middle layer of the cortex in the right hemisphere as calculated by freesurfer. Blue arrows point out primary sensory cortices (visual, auditory and sensory-motor cortex) which can be associated with increased R_1_ values. Red arrows point out regions of significant R_1_ differences from the ground truth prior to the B_1_^+^ correction, which are successfully corrected: on the coronal slice a strong left right asymmetry between similar cortical regions, while on the sagittal slice a strong anterior posterior variation is highlighted.

**Figure 8:**
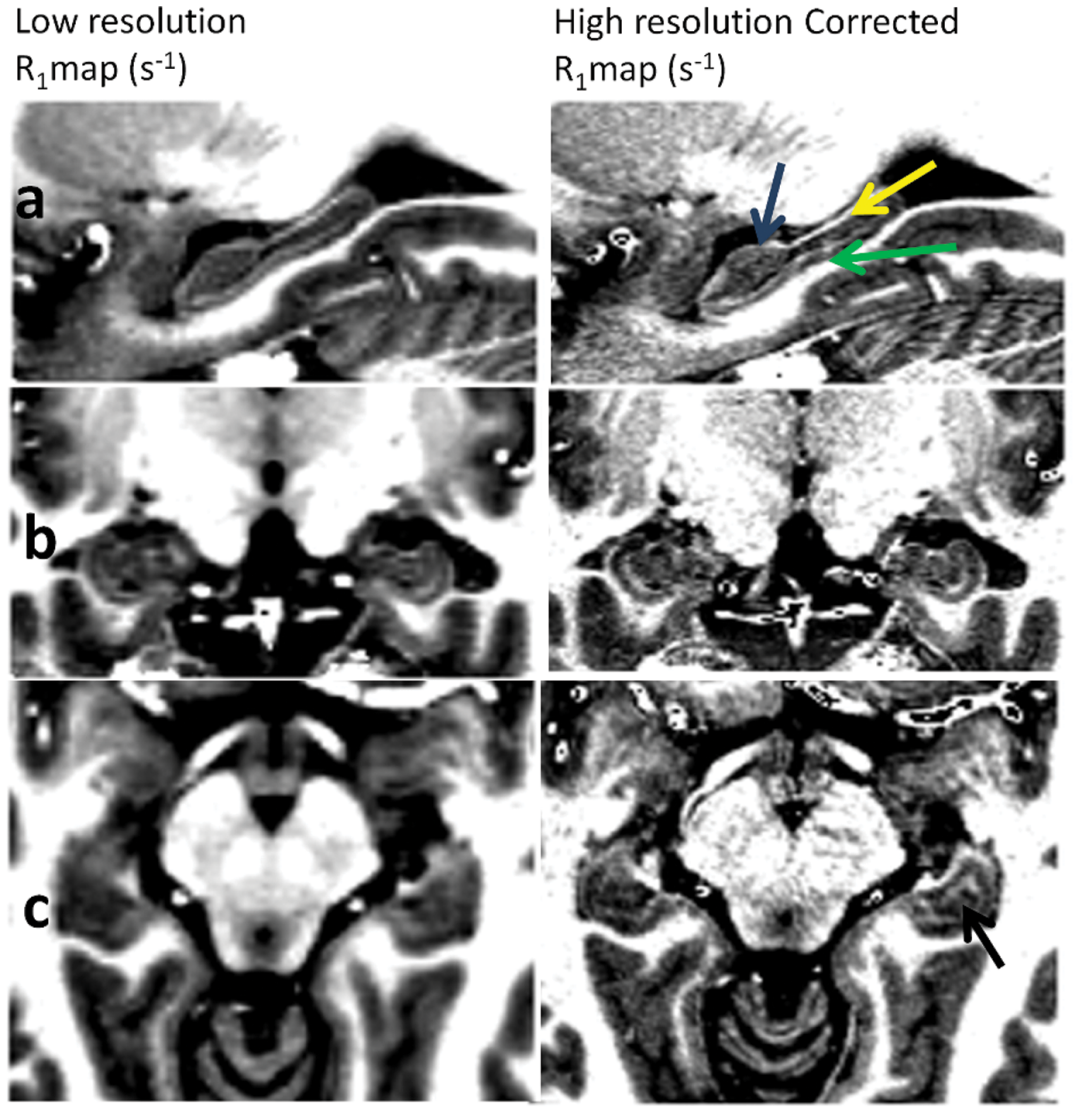
Transverse (a), sagittal (b) and coronal (c) slices covering the hippocampus of a high resolution corrected R_1_ map. Arrows show hippocampal structures: CA1, CA2 3 (blue arrow); fimbria of hippocampus (yellow arrow); CA4 and DG (black arrow); subiculum (green arrow) [32]. Such structures are only discernible in the high resolution dataset.

Figure 7 shows three different slices of R_1_ maps of the human brain (the range, 0.45–0.80 s^−1^, was set to enhance the variations observed within grey matter). In any of the R_1_ maps it is possible to observe the expected distribution of R_1_ in the cortex with higher R_1_ values being present on primary sensory areas (singled out by blue arrows), such as the sensory motor, auditory and visual cortex, with respect to other neighboring regions. Nevertheless, it is also possible to observe in Fig. 7b (uncorrected R_1_ maps) that many cortical inhomogeneities/asymmetries are observed (as pointed out by the red arrows) that are likely to be associated with the B_1_ inhomogeneity and are indeed removed when taking the B_1_ measurements into account (Fig. 7c). Figure 7d shows the inflated right hemisphere of an high resolution corrected R_1_ map of one single subject across the middle layer of the cortex. Blue arrows show the same cortical regions as those shown on Figure 7c, with the primary sensory areas clearly having increased R_1_ values when compared to the remaining cortex. Figure 8 shows an enlarged view of R_1_ maps in the hippocampal region demonstrating that the high resolution of the obtained R_1_ maps offers the possibility to use this quantitative information to visualize (the shape and separation between grey and white matter regions) and characterize its different fields and regions (with the dentate gyrus, DG, having the lowest R_1_ values, followed by the CA1–CA3 and finally the CA4 and subiculum having higher R_1_ values) [32]. The observation of the DG having a lower R_1_ value than the CA fields is in good agreement with recent histology literature [33]. A clear delineation of all these structures is only discernible in the high resolution dataset and its relaxometry properties could show changes preceding the changes in volume often observed in pathologies.

## Discussion

In this paper we have shown the ability to adapt the conventional T_1_ weighted protocol used for whole brain imaging at 7T to focus on the tissues whose T_1_’s lie in-between those of white and grey matter. Such adaptation allowed the visualization of thalamic nuclei and brain stem structures which were not visible in the conventional protocol because of lack of contrast to noise ratio (note that the observed contrast is still only related to the T_1_ of the different tissues). Particularly, the increased sensitivity to changes of grey and white matter T_1_ values could help detect or/and characterize early stages of idiophatic Parkinson disease [34] that have been associated with deposition of Lewy bodies in the medulla oblongata and structural changes that progress in a caudorostral pattern [35], [36], only affecting the substantia nigra in a stage where the first motor symptoms occur. Furthermore, the ability to recalculate a conventional T_1_-w contrast (where the contrast of subthalamic nuclei is still increased) could be used to obtain automatic segmentation of the whole brain, the thalamus and subsequently of its nuclei using available histology based atlas [37]. The better visualization or automatic delineation of some of these structures would allow a better localization of structures such as the such as the vim [6] which is of great importance in pre-surgery planning in pathologies such as in Parkinson’s disease or dystonia [8], [9].

In this work, it was demonstrated that the residual sensitivity to B_1_ inhomogeneities present in the R_1_ maps obtained with the MP2RAGE sequence can be removed using information from a B_1_ map (the choice of the B_1_ mapping technique is not crucial, and many other methods would be equally valid). In its current implementation, the B_1_ correction to the R_1_ maps was only considered to affect the excitation pulses and not the adiabatic inversion pulse, this could be introduced into the Bloch equation simulations by changing the inversion efficiency of the inversion pulse as a function of B_1_ and B_0_ map. This would be expected to have only a small effect on the R_1_ values measured as one of the main characteristics of the adiabatic pulse used is its low sensitivity to B_1_ and B_0_ variations once the adiabatic condition is achieved [38]. The success of the correction presented in this manuscript allows to obtain R_1_ maps in either: a reduced acquisition time (decreasing TR will now only have implication in terms of available contrast to noise [5]); increase contrast to noise ratio thanks to the possibility of using protocols with increased flip angles; increased resolution thanks to the possibility of using longer GRE blocks that would otherwise affect the B_1_ insensitivity of the method.

High spatial resolution R_1_ mapping at 3T has been shown to allow the identification of various cortical regions which have been validated by functional retinotopic and tonotopic studies [20], [39]. The presented protocol to obtain quantitative R_1_ values could be used in similar studies as there are remarkable similarities between the surface maps obtained (see Fig. 7d) with those found in literature, in which cortical regions were carefully validated with functional studies and the robustness of the methods were tested via test-retest of these regions in group studies. Although a comparison between the two methodologies is outside the scope of this paper (as the differences could result from the increased SNR, or R_1_ dispersion available at 7T, or from the different efficiency of the R1 mapping methodologies), it should be noted that the present study was acquired with a higher spatial resolution (0.65 mm vs 0.8 mm isotropic), was obtained in a shorter amount of time (12 mins vs 21 mins) and the through layer smoothing of the surface map shown on Fig. 7d was significantly smaller (2 mm vs 4 mm) than the cortical R_1_ maps shown in previous studies [20], [39]. Two considerations can be made from this observation: the proposed protocol has the potential to obtain a delineation of the primary sensory regions on individual subject data as shown by other groups; having a spatial resolution of the order of magnitude of the thickness of the different cortical layers (which have been demonstrated to have different relaxation properties) [7], makes the evaluation of R_1_ maps at different cortical depths meaningful, such extra information could help further differentiating cortical regions [7], [40] and will be the object of future research.

### Future Work and Conclusions

We have shown that, by optimizing the contrast of the MP2RAGE sequence to a specific range of T_1_’s at 7T, it is possible to gain access to clearer anatomical delineation of thalamic nuclei and brain stem structures. Such improved contrast could be an important asset in the context of automatic segmentation and in pre-surgical planning of Parkinson patients. One of the unanswered questions that will be the object of future work is the relationship between the observed sub-structures of the thalamus observed in T_1_-w imaging and the sub-thalamic nucleus identified with histology so that the automatic segmentation of the thalamus can be guided by the image contrast rather than simply the strength of the given prior.

We demonstrated the ability to obtain high resolution (0.65 mm isotropic) and high SNR R_1_ maps of the whole brain in ∼12 mins using the information from separately acquired R_1_ and B_1_ maps from the MP2RAGE and Sa2RAGE sequences. The quantitative R_1_ maps revealed subtle differences between distal GM tissues as shown in previous studies, but also within WM where R_1_ contrast is usually overlooked.

## Acknowledgments

The authors would like to thank Dr. Tobias Kober for the support in the implementation and maintenance of the sequences used throughout this study and Dr. Kieran O’Brien for having built the dielectric pads used to accentuate the B_1_ field variations in the scan-rescan experiment.
